# Evaluating the Implementation of the Connect for Health Pediatric Weight Management Program

**DOI:** 10.1001/jamanetworkopen.2023.52648

**Published:** 2024-01-25

**Authors:** Meg Simione, Holly M. Frost, Haley Farrar-Muir, Man Luo, Jazmin Granadeño, Carlos Torres, Alexy Arauz Boudreau, Jennifer Moreland, Jessica Wallace, Jackie Young, John Orav, Kerry Sease, Simon J. Hambidge, Elsie M. Taveras

**Affiliations:** 1 Department of Pediatrics, Mass General for Children, Boston, Massachusetts; 2 Department of Pediatrics, Harvard Medical School, Boston, Massachusetts; 3 Center for Health Systems Research, Office of Research, Denver Health and Hospital Authority, Denver, Colorado; 4 Department of Pediatrics, Denver Health and Hospital Authority, Denver, Colorado; 5 Department of Pediatrics, University of Colorado School of Medicine, Aurora; 6 Public Health Institute at Denver Health, Denver, Colorado; 7 Prisma Health, Greenville, South Carolina; 8 Department of Medicine, Brigham and Women’s Hospital, Boston, Massachusetts; 9 Department of Pediatrics, University of South Carolina School of Medicine, Greenville; 10 Ambulatory Care Services, Denver Health, Denver, Colorado

## Abstract

**Importance:**

Adoption of primary care interventions to reduce childhood obesity is limited. Progress in reducing obesity prevalence and eliminating disparities can be achieved by implementing effective childhood obesity management interventions in primary care settings.

**Objective:**

To examine the extent to which implementation strategies supported the uptake of research evidence and implementation of the Connect for Health pediatric weight management program.

**Design, Setting, and Participants:**

This quality improvement study took place at 3 geographically and demographically diverse health care organizations with substantially high numbers of children living in low-income communities in Denver, Colorado; Boston, Massachusetts; and Greenville, South Carolina, from November 2019 to April 2022. Participants included pediatric primary care clinicians and staff and families with children aged 2 to 12 years with a body mass index (BMI) in the 85th percentile or higher.

**Exposures:**

Pediatric weight management program with clinician-facing tools (ie, clinical decision support tools) and family-facing tools (ie, educational handouts, text messaging program, community resource guide) along with implementation strategies (ie, training and feedback, technical assistance, virtual learning community, aligning with hospital performance metrics) to support the uptake.

**Main Outcomes and Measures:**

Primary outcomes were constructs from the Reach, Effectiveness, Adoption, Implementation, Maintenance (RE-AIM) Framework examined through parent, clinician, and leadership surveys and electronic health record data to understand the number of children screened and identified, use of the clinical decision support tools, program acceptability, fidelity to the intervention and implementation strategies, and program sustainability.

**Results:**

The program screened and identified 18 333 children across 3 organizations (Denver Health, 8480 children [46.3%]; mean [SD] age, 7.97 [3.31] years; 3863 [45.5%] female; Massachusetts General Hospital (MGH), 6190 children [33.8%]; mean [SD] age, 7.49 [3.19] years; 2920 [47.2%] female; Prisma Health, 3663 children [20.0%]; mean [SD] age, 7.33 [3.15] years; 1692 [46.2%] female) as having an elevated BMI. The actionable flagging system was used for 8718 children (48%). The reach was equitable, with 7843 children (92.4%) from Denver Health, 4071 children (65.8%) from MGH, and 1720 children (47%) from Prisma Health being from racially and ethnically minoritized groups. The sites had high fidelity to the program and 6 implementation strategies, with 4 strategies (67%) used consistently at Denver Health, 6 (100%) at MGH, and 5 (83%) at Prisma Health. A high program acceptability was found across the 3 health care organizations; for example, the mean (SD) Acceptability of Intervention Measure score was 3.72 (0.84) at Denver Health, 3.82 (0.86) at MGH, and 4.28 (0.68) at Prisma Health. The implementation strategies were associated with 7091 (39%) uses of the clinical decision support tool. The mean (SD) program sustainability scores were 4.46 (1.61) at Denver Health, 5.63 (1.28) at MGH, and 5.54 (0.92) at Prisma Health.

**Conclusions and Relevance:**

These findings suggest that by understanding what strategies enable the adoption of scalable and implementation-ready programs by other health care organizations, it is feasible to improve the screening, identification, and management of children with overweight or obesity and mitigate existing disparities.

## Key Points

**Question:**

What is the extent to which implementation strategies can support the adoption of a pediatric weight management program in primary care?

**Findings:**

In this quality improvement study of 18 333 children at 3 US health care organizations, implementation strategies were found to be helpful in promoting program adoption and equitable reach. The program was highly acceptable by clinicians, well-liked by families, and determined to have factors that would support program sustainability.

**Meaning:**

These findings suggest that understanding how to implement programs in primary care may increase the uptake of evidence-based programs for children with overweight and obesity.

## Teaser

This quality improvement study examines the implementation of the Connect for Health pediatric weight management program among 3 health care organizations with high numbers of children living in low-income communities.

## Introduction

Childhood obesity remains at historically high prevalence, and racial, ethnic, and socioeconomic disparities continue to widen.^1,2,3,4^ During the COVID-19 pandemic, obesity rates increased,^5,6^ necessitating evidence-based interventions. Adoption of primary care interventions to reduce obesity remains limited. Additionally, children from racially and ethnically minoritized groups continue to be disproportionately affected by obesity, indicating that they may not be benefiting equally from obesity prevention efforts. Progress in reducing obesity prevalence and eliminating disparities can be achieved by implementing effective childhood obesity management interventions in primary care settings.^7,8^ Connect for Health helps to fill that gap by delivering an evidence-based pediatric weight management program for systemwide delivery that leverages community and clinical resources to improve the quality of clinical care for childhood obesity.^9,10,11^ In a randomized clinical trial (RCT), Connect for Health improved child body mass index (BMI; calculated as weight in kilograms divided by height in meters squared) and family-centered outcomes, including child health-related quality of life and parental empowerment.^10,11^ Despite programs that have shown effectiveness, few programs have been implemented and studied in primary care settings due to a myriad of barriers, including shortage of resources, insurance coverage and reimbursement, program sustainability, and low prioritization of child health, which is critical to moving evidence from an RCT to routine practice to improve care for children and mitigate disparities.^7,12^

This study aims to promote and facilitate the uptake of research evidence generated by the Connect for Health trial in 3 health care organizations with substantially high numbers of children living in low-income communities. We aimed to examine the extent to which our implementation strategies supported the implementation of the Connect for Health program at participating health care organizations using the Reach-Adoption-Effectiveness-Implementation-Maintenance (RE-AIM) Framework. Based on prior work with our stakeholders, we hypothesized that the program would be successfully implemented with minor adaptations needed for local context.

## Methods

This report follows the Standards for Quality Improvement Reporting Excellence (SQUIRE 2.0) reporting guideline.^19^ The Mass General Brigham Institutional Review Boards approved this study, and it was registered at ClinicalTrials.gov (NCT0212446). For clinician and parent surveys, informed consent was included within the surveys and completion of the surveys documented consent. Given the size and scope of the data pull, a waiver of consent was granted for the electronic health record data. 

Connect for Health is a pediatric weight management program that aligns with the United States Preventive Services Task Force and the American Academy of Pediatrics recommendations regarding screening, comprehensive evaluation, and behavior change counseling.^9,10,11,13,14,15^ Prior to implementing the program, we engaged stakeholders (including families, clinicians, and hospital leadership) to adapt the program and refine the implementation strategies guided by the Consolidated Framework for Implementation Research.^16^ We used organization-specific stakeholder input to determine how the organizations would implement and customize the program to meet their local context.^17^ We further adapted the program during the implementation period in response to the COVID-19 pandemic to account for a shift to virtual visits and other competing demands.^18^

We partnered with health care organizations that predominately care for children from low-income communities: Denver Health in Denver, Colorado; Massachusetts General Hospital (MGH) in Boston, Massachusetts; and Prisma Health in Greenville, South Carolina. They were selected because they had pediatric or family medicine practices based in hospitals, federally qualified health centers or community health centers, served a low-income population with individuals from racially and ethnically minoritized groups, had high rates of obesity, and used Epic as their electronic health record (EHR) platform. The program aimed to improve the care of children ages 2 to 12 years with an elevated BMI. Each organization defined an elevated BMI as being in the 85th percentile or greater or in the 95th percentile or greater. Implementation periods were December 2019 to April 2021 at Denver Health, December 2019 to April 2022 at MGH, and November 2019 to April 2022 at Prisma Health. Implementation length varied due to organization-specific policies and practices as a result of the COVID-19 pandemic.

### Connect for Health Program and Implementation Strategies

The Connect for Health program and implementation strategies have been described previously.^9,17,20^ Briefly, the program consists of family materials that support behavioral change and connect families to resources available in English, Spanish, and Haitian Creole. They include printable family educational materials, social and community-informed text messages, and community resource guides to identify resources within the family’s neighborhoods. The clinician tools include a flagging system, known as Best Practice Alert (BPA), to identify children with elevated BMIs and a note template, known as a Smart Set, which guides clinicians in best management practices. The program is intended to be delivered by medical clinicians in primary care during well-child visits and can be used as deemed clinically appropriate. It is integrated into existing workflows to support best management practices and provide educational materials and resources for behavior change. The organizations customized the program to suit their workflow and needs. For example, Denver Health uses a team-based care approach and had the flagging system alert medical assistants who would initiate enrollment in the text messaging program and laboratory orders, whereas physicians at MGH and Prisma Health received a noninterruptive BPA, interacted with the clinical decision support tools, enrolled in text messaging, and provided educational materials.

The implementation strategies to facilitate the adoption of the program were based on the Expert Recommendations for Implementing Change,^21^ designed to promote the equitable uptake of Connect for Health and delivered by each organization. The strategies were tailored based on stakeholder engagement and included: (1) conducting ongoing clinician trainings; (2) providing local technical assistance and consultation; (3) creating a virtual learning community (centralized delivery); (4) aligning with organization performance metrics; (5) providing feedback to clinicians and staff; and (6) providing practice facilitation.

### RE-AIM Framework Evaluation

The evaluation of the multisite implementation study was guided by the RE-AIM Framework.^22^ Table 1 describes the RE-AIM constructs and how we operationalized them. Data collection included surveying of parents, clinicians, and hospital leadership; abstracting clinical data from the EHR; and collecting utilization data of the EHR tools.

**Table 1.** Application of the RE-AIM Framework to the Evaluation of the Connect for Health Program

RE-AIM construct	Evaluation measure	Denver Health	MGH	Prisma Health
Reach	Child sociodemographic characteristics, No. (%)			
	From racially or ethnically minoritized groups^a^	7843 (92.4)	4071 (65.8)	1720 (47.0)
	Public insurance	7657 (90.3)	3983 (64.4)	516 (14.1)^b^
	No. of children in which action was taken on the Best Practice Alert/No. of children screened and identified with an elevated BMI using the flagging system (ie, Best Practice Alert), (%)	5073/8480 (60)	2545/6190 (41)	1100/3663 (30)
Adoption	Setting-level characteristics: No. of practices and practice types	10 Practices; 19 school-based health clinics (pediatric; family medicine; school-based health clinic)	6 Practices (pediatric; family medicine; internal medicine-pediatrics)	8 Practices (pediatric)
	Staff-level characteristics, No. of clinicians and team members and their roles	283 Clinicians (physician; physician assistant, nurse practitioner); 148 medical assistants	59 Clinicians (physician)	47 Clinicians (physician; physician assistant, nurse practitioner)
	Total smart set utilizations for unique patients, No. (%)	4938 (58)	1007 (16)	1146 (31)
	Total text messaging orders for unique patients, No. (%)	4553 (54)	1074 (17)	423 (12)
Implementation				
Acceptability	Acceptability of Intervention Measure, mean (SD) score^c^	3.72 (0.84)	3.82 (0.86)	4.28 (0.68)
	Connect for Health program acceptability, mean (SD) score^c^	3.64 (0.67)	3.47 (0.53)	4.00 (0.39)
	Connect for Health telehealth acceptability, mean (SD) score^c^	Telehealth adaptations not implemented	3.36 (0.51)	3.87 (0.66)
Fidelity	Program fidelity	Minor modifications	Minor modifications	Minor modifications
	Implementation strategies used consistently, No./total No. (%)	4/6 (67)	6/6 (100)	5/6 (83)
Maintenance	Sustainability survey, mean (SD) score^d^	4.46 (1.61)	5.63 (1.28)	5.54 (0.92)
	Reach over time, No. of children with action taken/total No. of children screened and identified, (%)^e^	285/309 (92)	361/1015 (36)	37/568 (7)
	Adoption over time total smart set utilizations for unique patients, No. (%)^e^	249 (81)	85 (8)	41 (7)

Abbreviation: BMI, body mass index.

^a^ Includes children identifying as African American or Black, non-Hispanic; American Indian or Alaskan Native; Asian; Hispanic or Latino; or Native Hawaiian or Pacific Islander. The No. (%) of race and ethnicity data can be found in Table 2.

^b^ Insurance was unreported for 2779 (75.9) children.

^c^ Item score range 1 to 5. Higher scores indicate greater acceptability.

^d^ Item score range 1 to 7. Higher scores indicate greater sustainability. Questions were adapted from the Clinical Sustainability Assessment Tool and the Program Sustainability Assessment Tool.

^e^ The maintenance period was 3 months.

#### Reach

To understand the number of children identified and screened by the program, we determined the number of BPAs for unique children during the implementation period (and thereby considered eligible to receive the program) and further determined the number of unique children in which action was taken on the BPA (BPAs were actionable and could, for example, add a diagnosis code). In addition, we abstracted child sociodemographic data from the EHR to understand the equitable reach of the program.^23^

#### Effectiveness

Effectiveness was defined as the impact of a program on individual outcomes and broader impacts. We surveyed a representative sample of parents following a well-child visit in which the BPA was sent to understand their experiences with the program. Survey items included close-ended questions about the program components, experiences with care,^24^ and impacts of the COVID-19 pandemic.^25^ The survey was conducted via phone (or email upon request) and was available in English and Spanish.

We also examined changes in child BMI *z* scores by comparing the pattern of BMI *z* score changes in children who were eligible to receive the program (BMI greater than or equal to the 85th percentile or 95th percentile) to control children who were not eligible for the program (BMI between the 50th to 84th percentile or 50th to 94th percentile) from the same institutions. In addition, we used a second control group of children with elevated BMIs at geographically and demographically (ie, city characteristics, race, ethnicity, insurance) matched community health centers allowing us to compare BMI *z* score changes. We collected multiple height and weight measurements from the EHR and calculated age-specific and sex-specific BMI *z* scores at approximately 12 months and 24 months (24-month BMI *z* scores were collected for MGH and Prisma Health and their geographic controls due to the extended implementation period).^26^

#### Adoption

We collected setting (ie, type and number of practices) and staff level (ie, number of clinicians and roles) characteristics for the organizations. Smart Set utilizations and text messaging referral orders were also collected and abstracted through the EHR to understand program adoption.

#### Implementation

The implementation support team, including clinician champions, practice coaches, and project managers, completed checklists to measure fidelity to the program and implementation strategies during late implementation.^27,28^ Clinicians and medical assistants completed surveys about program acceptability using the Acceptability of Intervention Measure,^29^ and additional items about the acceptability of individual program components and telehealth adaptations during mid to late implementation. The survey responses options ranged from 1 to 5 with high scores indicating greater acceptability.

#### Maintenance

The maintenance period was defined as a 3-month period as organizational implementation supports and research funding were being reduced. Leadership and key clinicians involved with program implementation completed surveys about the sustainability of the program using items from the Clinical Sustainability Assessment Tool^30^ and the Program Sustainability Assessment Tool,^31^ which evaluated factors, such as stakeholder engagement, organizational readiness, workflow integration, training, and evidence of effectiveness that would support program sustainability. The survey response options ranged from 1 to 7 with high scores indicating a greater likelihood of sustainability. We also determined the reach and adoption of the program during this period.

### Statistical Analysis

We analyzed the family experience of care and clinician and leadership survey data and EHR utilization data using descriptive statistics, including frequency and percentage or mean (SD). We examined changes in child BMI *z* scores using a regression discontinuity design^32^ comparing children eligible to receive the program to projected BMI *z *score changes based on control children who were not eligible for the program. We also compared children eligible with receive the program to children from geographically and demographically matched community health centers. We calculated changes in BMI *z *scores after multiple imputations of any missing follow-up data, based on baseline and preimplementation BMI *z *scores and sociodemographic characteristics. We used a segmented regression model with BMI *z *score change as the outcome and baseline BMI percentile as the exposure to estimate the pattern and adjusted for sociodemographic factors (age, sex, race or ethnicity, insurance, baseline BMI). A linear regression model was used to estimate the pattern for children from the matched community health centers and to compare the observed BMI *z *score changes in the corresponding organizations. In all analyses, we used a 2-sided alpha level of .05 to test for statistical significance. R Studio Software (version 4.1.0) was used for the statistical analyses. Data were analyzed from March 2022 to December 2022.

## Results

### Reach

In this study, the BPA resulted in the screening and identification of 18 333 children across 3 organizations (Denver Health, 8480 children [46.3%]; mean [SD] age, 7.97 [3.31] years; 3863 [45.5%] female; MGH, 6190 children [33.8%]; mean [SD] age, 7.49 [3.19] years; 2920 [47.2%] female; Prisma Health, 3663 children [20.0%]; mean [SD] age, 7.33 [3.15] years; 1692 [46.2%] female) as having an elevated BMI, and action was taken on the BPA for 8718 children (48%). Table 1 shows the reach by organization. As intended, the program reached children who were from low-income families (7657 [90.3]% had public insurance at Denver Health and 3983 [64.4%] had public insurance at MGH; limited insurance data were available at Prisma Health) and racially and ethnically diverse with 7843 (92.4%) from Denver Health, 4071 (65.8%) from MGH, and 1720 (47%) from Prisma being from racially and ethnically minoritized groups (Table 2 and eTable 1 in Supplement 1).

**Table 2.** Characteristics of Children Who Were Screened and Identified by the Connect for Health Program^a^

Characteristics	Participant, No. (%)		
	Denver Health (n = 8480)	MGH (n = 6190)	Prisma Health (n = 3663)
Age, mean (SD), y	7.97 (3.31)	7.49 (3.19)	7.33 (3.15)
BMI, mean (SD), kg/m2	22.64 (4.72)	22.11 (4.48)	23.91 (4.72)
BMI *z *score, mean (SD)	1.84 (0.56)	1.82 (0.59)	2.24 (0.49)
Sex			
Female	3863 (45.5)	2920 (47.2)	1692 (46.2)
Male	4617 (54.5)	3270 (52.8)	1971 (53.81)
Race and ethnicity			
African American or Black, Non-Hispanic	1038 (12.2)	606 (9.8)	491 (13.4)
American Indian or Alaska Native, Native Hawaiian or Pacific Islander, Asian	349 (4.1)	654 (10.6)	29 (0.8)
Hispanic or Latino	6456 (76.1)	2811 (45.4)	1200 (32.8)
White, Non-Hispanic	594 (7.0)	1457 (23.5)	1194 (32.6)
Not reported	43 (0.5)	662 (10.7)	749 (20.4)
Language			
English	4550 (53.7)	3922 (63.7)	Not reported
Spanish	3486 (41.1)	1823 (29.6)	Not reported
Missing	1 (XX%)	32 (XX%)	NA
Other	443 (5.2)	413 (6.71)	Not reported
Insurance type			
Public	7657 (90.3)	3983 (64.4)	516 (14.1)
Private	823 (9.7)	2205 (35.6)	368 (10.0)
Not reported	0	0	2779 (75.9)
BMI category			
Overweight	3572 (42.1)	2766 (44.7)	NA
Obesity	3350 (39.5)	2341 (37.8)	2511 (68.6)
Severe obesity	1558 (18.4)	1083 (17.5)	1152 (31.4)

Abbreviation: BMI, body mass index (calculated as weight in kilograms divided by height in meters squared); MGH, Massachusetts General Hospital.

^a^ Denver Health and MGH reached children with a BMI in the 85th percentile or higher and Prisma Health reached children with a BMI in the 95th percentile or higher.

### Effectiveness

**Figure 1.**
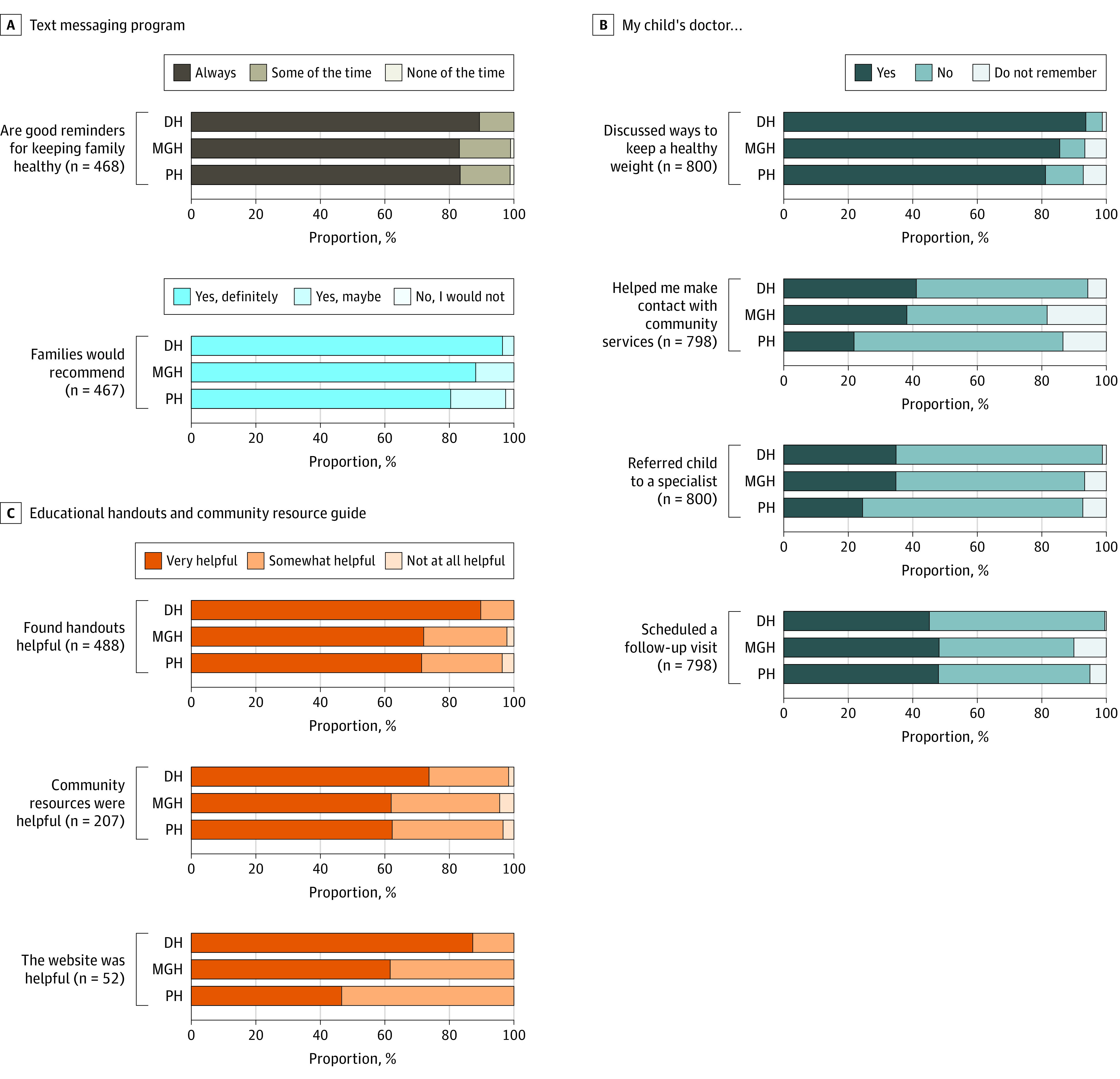
Results Evaluating the Connect for Health Program and Experiences With Care Items were only asked if parents affirmed receiving the program component. Three hundred surveys were completed at Denver Health (DH) from February 26 to September 2, 2021; 300 surveys were completed at Massachusetts General Hospital (MGH) from October 14, 2021, to August 19, 2022; and 202 surveys were completed at Prisma Health (PH) from July 16, 2021, to June 20, 2022.

We surveyed 802 parents of children with elevated BMIs. More than 80% of parents reported the text messaging program served as a good reminder to keep their family healthy (Denver Health, 139 [89.7%]; MGH, 150 [83.3%]; Prisma, 111 [83.5%]), endorsed recommending the program to families or friends (Denver 149 [96.8%]; MGH, 159 [88.3%]; Prisma, 107 [80.5%]), and reported that their child’s clinician discussed weight management at their well-child visit (Denver Health, 283 [94.3%]; MGH, 257 [86.2%]; Prisma, 167 [81.7%]) (Figure 1 and eTable 1 in Supplement 2). Despite the program’s focus on behavioral change, families reported net negative impacts on health behaviors, possibly due to the COVID-19 pandemic and related policies that the program was unable to offset (eTable 2 in Supplement 2).

**Figure 2.**
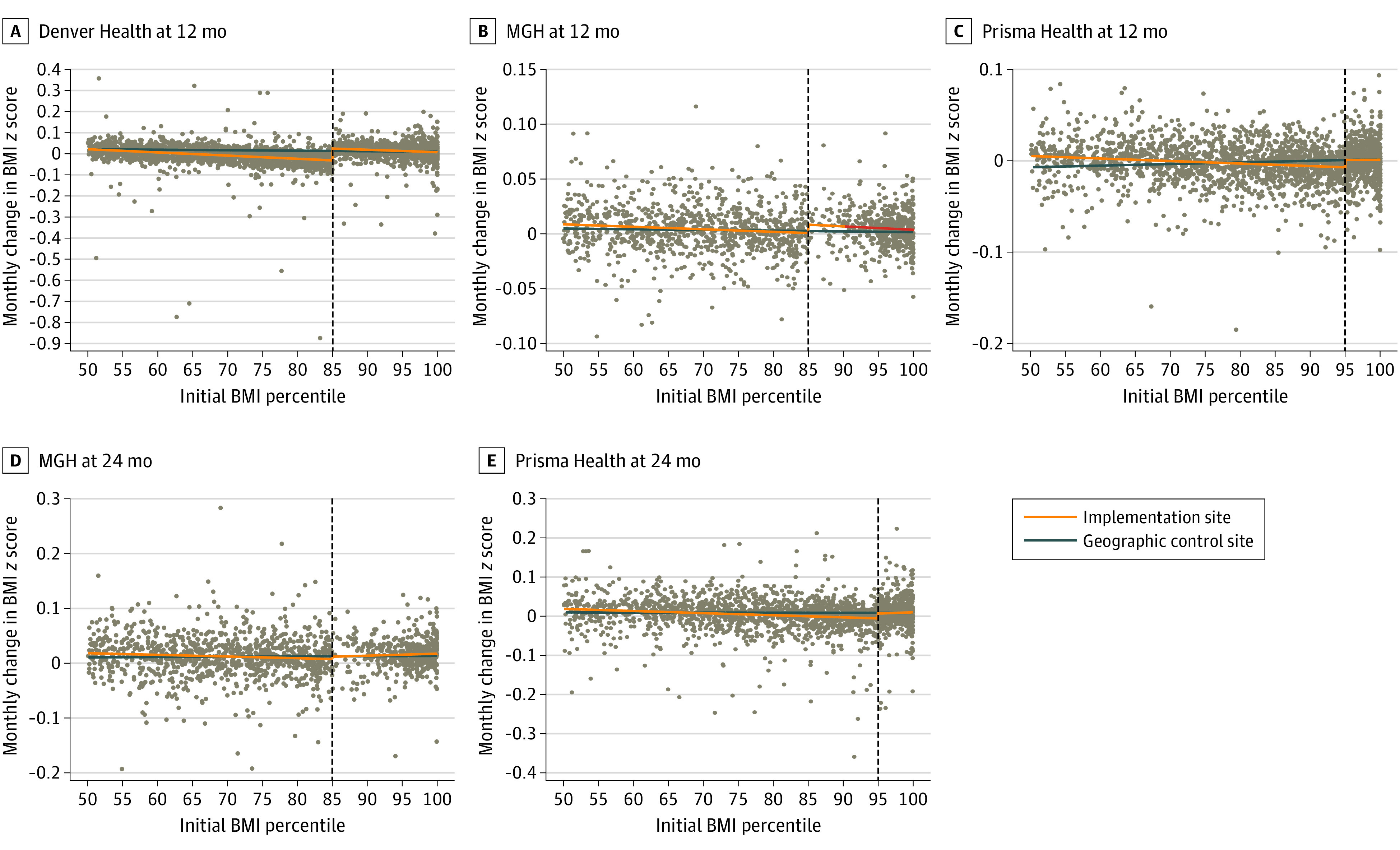
Changes in Body Mass Index (BMI) for Children Eligible for the Program and Controls Massachusetts General Hospital (MGH) and Prisma Health had extended implementation periods and Denver Health did not. At sites implementing Connect for Health, children eligible to receive the program had a BMI (calculated as weight in kilograms divided by height in meters squared) in the 85th percentile or higher (Denver Health and MGH) or 95th percentile or higher (Prisma Health). Children who were not eligible for the program were controls if they had a BMI between the 50th and 84th percentiles at Denver Health and MGH or a BMI between the 50th and 94th percentile at Prisma Health. Eligible children at geographically and demographically matched organizations had a BMI in the 50th percentile or higher.

The results of the monthly BMI *z *score change evaluation are shown in Figure 2, and the results of the segmented regression for the children eligible to receive the program and the control children are shown in eTable 3 in Supplement 2. Overall, we found increases in BMI *z* scores for children eligible to receive the program across the 3 organizations. At 12 months, the BMI of children who were eligible to receive the program was associated with a 0.16 (95% CI, 0.15 to 0.17) mean change at Denver Health; 0.17 (95% CI, 0.15 to 0.20) mean change at MGH; and 0.09 (95% CI, 0.07 to 0.11) mean change at Prisma Health. At 24 months, the BMI of children who were eligible to receive the program was associated with a 0.10 (95% CI, 0.07 to 0.14) mean change at MGH and 0.02 (95% CI, −0.01 to 0.05) mean change at Prisma Health. In comparison to the geographically and demographically matched community health centers, we saw no improvements in BMI change (eTable 4 in Supplement 2).

### Adoption

The practices that adopted the program included pediatric primary care, family medicine, internal medicine pediatrics, and school-based health clinics; and clinicians, such as physicians, nurse practitioners, physician assistants, and medical assistants, used the program. Across the organizations, the Smart Set was used 7091 times (39% of children who were reached) and 6152 text messaging orders were placed (33%); however, the rates of uptake varied by organization (Table 1). We could not determine if patients who did not participate in the text messaging program were because it was not offered by clinicians or if patients declined participation.

### Implementation

We examined the fidelity and acceptability to understand program implementation (Table 1). We found that the sites had high fidelity to the program and 6 implementation strategies, with 4 strategies (67%) used consistently at Denver Health, 6 (100%) at MGH, and 5 (83%) at Prisma Health. Modifications were made after the program was implemented, mostly related to the COVID-19 pandemic (for example, ensuring the BPA worked for telehealth visits). We found high program acceptability across the 3 health care organizations as evidenced in the survey scores. For example, the mean (SD) Acceptability of Intervention Measure score was 3.72 (0.84) at Denver Health, 3.82 (0.86) at MGH, and 4.28 (0.68) at Prisma Health.

### Maintenance

We found high scores on the sustainability survey indicating that the sites during the preimplementation and implementation phases of the project had addressed factors that would support program sustainability. The mean (SD) program sustainability scores were 4.46 (1.61) at Denver Health, 5.63 (1.28) at MGH, and 5.54 (0.92) at Prisma Health. When examining the reach and continued uptake of the program during a 3-month maintenance period (Table 1), we found the reach to increase for Denver Health (285 of 309 children [92%]), remain approximately the same for MGH (36 of 1015 children [36%]), and decrease for Prisma Health (37 of 568 children [7%]). Overall, the program continued to be used as support and project funding was decreased, and sites were cognizant about addressing factors to support program sustainability.

## Discussion

Using the RE-AIM Framework, we evaluated the implementation of the Connect for Health program to fill the gap in evidence-based pediatric weight management programs in the primary care setting. We found the implementation strategies to be helpful in promoting program adoption and equitable reach. The program was highly acceptable by clinicians and well-liked by families and determined to have factors that would support program sustainability, although it did not improve child BMI. The success of program implementation was attributed to the organization-specific stakeholder engagement that occurred prior to and during implementation.

Despite weight management programs that have been shown to improve health outcomes and primary care being a suitable setting for such interventions, few programs have been implemented and systematically evaluated in primary care.^33,34^ Several studies are currently ongoing with results pending,^35,36^ and similar to our evaluation, the studies are using implementation science frameworks to understand the adoption, reach, implementation, and sustainability. Given the high prevalence and the limited access health care organizations have to childhood obesity programs,^8,12^ utilizing implementation science frameworks to implement interventions is crucial to closing the research-to-practice gap. The frameworks allow for an understanding of barriers and facilitators so that programs and implementation strategies can be adapted to suit local contexts.^7,37^

During program implementation, the health care organizations faced barriers and challenges. Without having undertaken the planning prior to program implementation and stakeholder engagement, the organizations may not have been able to address barriers and pivot during the COVID-19 pandemic, for example, by adapting Connect for Health for telehealth.^18,38,39^ The high program acceptability by clinicians, staff, and families across the 3 organizations was attributable to the stakeholder engagement that sought input regarding the family educational materials, clinical decision support tools and workflow, and implementation strategies.^17^ The broad and equitable reach of the program was also attributable to stakeholder engagement, leading to tailored cultural adaptations and clinician and staff training that emphasized childhood obesity disparities, best management practices, and program use.^17^ Planning for implementation and stakeholder engagement have consistently been shown to be essential when implementing clinical innovations, and others implementing childhood obesity interventions should incorporate a planning period prior to implementation and engage stakeholders to ensure successful adoption.^38,39^

The health care organizations also addressed sustainability early in the process. An important aspect of sustainability planning is acknowledging that program adaptations will be needed,^40^ and the organizations were encouraged to customize Connect for Health.^17^ During the maintenance period, we found differences in the program reach and adoption across the organizations, which highlighted the need for ongoing monitoring and adapting of programs as they move into a sustainability phase. For example, Prisma Health’s low uptake of the EHR tools is explained by a system-level EHR change to well-child visits which made it difficult to see the flag that will now need to be modified to support continued use.

We also found variation in the uptake of the EHR tools among the health care organizations throughout the implementation period. Denver Health’s high uptake was likely due to their approach of having medical assistants primarily interact with the tools. MGH and Prisma Health had physicians interact with the tools, and their uptake is consistent with the literature regarding the adoption of clinical decision-support tools.^41^ Denver Health’s high uptake supported the importance of a team-based care approach, stakeholder engagement, and providing clinician and staff training, which they did extensive engagement and training.^20^

The previous Connect for Health RCT found that the clinical decision support tools, family educational materials, and text messaging program improved child BMI.^11^ In this study, we did not find a change in child BMI, which may be explained by the COVID-19 pandemic and its resultant impacts and related policies. Research has shown that child BMIs increased and behaviors that support healthy lifestyles were affected during the COVID-19 pandemic.^5,6^ We also observed this change in health behaviors as evidenced by the results of the family survey questions.

### Limitations

This study has limitations. The implementation sites were all large health care organizations. Although the organizations had community health centers and federally qualified health clinics, the implementation in health centers that were not affiliated with academic health centers may be different. The study was intended to be a pragmatic trial, and we had no true control arm. We also did not have additional outcomes regarding changes in health behaviors as the intent was to collect readily available data from the EHR.

## Conclusions

The strategies used to implement the Connect for Health pediatric weight management program were found to be effective in promoting adoption, broad and equitable reach, and addressing the sustainability of the program. The program was found to be acceptable by clinicians and families. Connect for Health is a scalable, implementation ready program that can be adopted by other health care organizations to improve the screening, identification, and management of children with overweight and obesity and mitigate existing disparities. Understanding how to implement programs in primary care will increase the uptake and sustainability of evidence-based weight management programs thereby improving the care of children with overweight and obesity.

## Notes

Supplement 1. **eTable 1.** Comparisons of Sociodemographics of Children Who Had Action Taken on the Best Practice Alert (BPA) and Those Who Did Not **eTable 2.** Survey Results Evaluating Family’s Experiences With the Connect for Health Program **eTable 3.** Segmented Regression Model of Body Mass Index (BMI) *Z* Score Changes by BMI Percentiles in 5 Percentile Increments of Organizations Implementing Connect for Health **eTable 4.** Segmented Regression Model of Body Mass Index (BMI) *Z* Score Changes By BMI Percentiles in 5 Percentile Increments of Geographically and Demographically Matched Control Organizations

Supplement 2.
Data Sharing Statement
