# Supplementary material for: Evaluating the Implementation of the Connect for Health Pediatric Weight Management Program
Source: JAMA Netw Open. 2024 Jan 25;7(1):e2352648. doi: 10.1001/jamanetworkopen.2023.52648 (PMC10811559; doi:10.1001/jamanetworkopen.2023.52648)
Supplement: Supplement 1. — eTable 1. Comparisons of Sociodemographics of Children Who Had Action Taken on the Best Practice Alert (BPA) and Those Who Did Not eTable 2. Survey Results Evaluating Family’s Experiences With the Connect for Health Program eTable 3. Segmented Regression Model of Body Mass Index (BMI) Z Score Changes by BMI Percentiles in 5 Percentile Increments of Organizations Implementing Connect for Health eTable 4. Segmented Regression Model of Body Mass Index (BMI) Z Score Changes By BMI Percentiles in 5 Percentile Increments of Geographically and Demographically Matched Control Organizations [file jamanetwopen-e2352648-s001.pdf]

Supplemental Online Content

Simione M, Frost HM, Farrar-Muir H, et al. Evaluating the implementation of the Connect for Health pediatric weight management program. *JAMA Netw Open*. 2023;7(1):e2352648. doi:10.1001/jamanetworkopen.2023.52648

eTable 1. Comparisons of Sociodemographics of Children Who Had Action Taken on the Best Practice Alert (BPA) and Those Who Did Not

eTable 2. Survey Results Evaluating Family's Experiences With the Connect for Health Program

eTable 3. Segmented Regression Model of Body Mass Index (BMI) *Z* Score Changes by BMI Percentiles in 5 Percentile Increments of Organizations Implementing Connect for Health

eTable 4. Segmented Regression Model of Body Mass Index (BMI) Z Score Changes By BMI Percentiles in 5 Percentile Increments of Geographically and Demographically Matched Control Organizations

This supplemental material has been provided by the authors to give readers additional information about their work.

© 2023 Simione M et al. *JAMA Network Open.*

eTable 1. Comparisons of Sociodemographics of Children Who Had Action Taken on the Best Practice Alert (BPA) and Those Who Did Not

**Denver Health**			
	**Action taken on the BPA (N=5073)**	**Action not taken on the BPA (N=3407)**	
	**Mean (SD)**	**Mean (SD)**	**p-values**
**Age**	8.28 (3.29)	7.49 (3.29)	**<0.001**
**BMI**	23.31 (4.95)	21.65 (4.15)	**<0.001**
**BMI Z-Score extended**	1.87 (0.61)	1.73 (0.60)	**<0.001**
	**N(%)**	**N(%)**	
**Child sex**			
Female	2264 (44.63)	1599 (46.93)	**0.04**
Male	2809 (55.37)	1808 (53.07)	
**Race/ Ethnicity**			
African American or Black, Non-Hispanic	613 (12.08)	425 (12.47)	**<0.001**
Asian, Non-Hispanic	128 (2.52)	115 (3.38)	
Hispanic/Latino	3948 (77.82)	2508 (73.61)	
White, Non-Hispanic	303 (5.97)	291 (8.54)	
Other	56 (1.10)	50 (1.47)	
Not Reported	25 (0.49)	18 (0.53)	
**Language (missing n=1)**			
English	2665 (52.53)	1885 (55.33)	**<0.001**
Spanish	2178 (42.93)	1308 (38.39)	
Other	230 (4.53)	213 (6.25)	
**Insurance Type**			
Public	4617 (91.01)	3040 (89.23)	**0.007**
Private	456 (8.99)	367 (10.77)	
**BMI Category**			
Overweight	1822 (35.92)	1750 (51.36)	**<0.001**
Obesity	2158 (42.54)	1192 (34.99)	
Severe Obesity	1093 (21.55)	465 (13.65)	

© 2023 Simione M et al. *JAMA Network Open.*

MGH			
	**Action taken on the BPA (N=2545)**	**Action not taken on the BPA (N=3645)**	
	**Mean (SD)**	**Mean (SD)**	**p-values**
**Age**	7.55 (3.05)	7.45 (3.28)	0.23
**BMI**	22.57 (4.52)	21.79 (4.43)	**<0.001**
**BMI z-score extended**	1.90 (0.69)	1.73 (0.62)	**<0.001**
	**N(%)**	**N(%)**	
**Child sex**			
Female	1239 (48.68)	1681 (46.12)	**0.047**
Male	1306 (51.32)	1964 (53.88)	
**Race/ Ethnicity**			
African American or Black, Non-Hispanic	257 (10.10)	349 (9.57)	**<0.001**
Asian, Non-Hispanic	111 (4.36)	162 (4.44)	
Hispanic/Latino	1241 (48.76)	1570 (43.07)	
White, Non-Hispanic	514 (20.20)	943 (25.87)	
Other	164 (6.44)	217 (5.95)	
Not Reported	258 (10.14)	404 (11.08)	
**Language (missing n=32)**			
English	1473 (58.27)	2449 (67.47)	**<0.001**
Spanish	848 (33.54)	975 (26.86)	
Other	207 (8.19)	206 (5.67)	
**Insurance Type (missing n=2)**			
Public	1753 (68.88)	2230 (61.21)	**<0.001**
Private	792 (31.12)	1413 (38.79)	
**BMI Category**			
Overweight	945 (37.13)	1821 (49.96)	**<0.001**
Obesity	1043 (40.98)	1298 (35.61)	
Severe Obesity	557 (21.89)	526 (14.43)	

© 2023 Simione M et al. *JAMA Network Open.*

Prisma Health			
	Action taken on the BPA (N=1100)	Action not taken on the BPA (N=2563)	
	**Mean (SD)**	**Mean (SD)**	**p-values**
**Age**	7.72 (2.93)	7.16 (3.22)	**<0.001**
**BMI**	24.79 (2.95)	23.54 (4.57)	**<0.001**
**BMI Z-Score extended**	2.29 (0.73)	2.15 (0.61)	**<0.001**
	**N(%)**	**N(%)**	
**Child sex**			
Female	527 (47.91)	1165 (45.45)	0.17
Male	573 (5.09)	1398 (54.55)	
**Race/ Ethnicity**			
African American or Black, Non-Hispanic	178 (16.2)	313 (12.2)	**0.002**
Asian, Non-Hispanic	5 (0.5)	15 (0.6)	
Hispanic/Latino	372 (33.8)	828 (32.3)	
White, Non-Hispanic	344 (31.3)	850 (33.2)	
Other	5 (0.5)	4 (0.2)	
Not Reported	196 (17.8)	553 (21.6)	
**Insurance Type**			
Public	144 (13.1)	372 (14.5)	0.52
Private	111 (10.1)	257 (10.0)	
Not Reported	845 (76.8)	1934 (75.5)	
**BMI Category**			
Obesity	657 (59.7)	1854 (72.3)	**<0.001**
Severe Obesity	443 (40.3)	709 (27.7)	

Note. BPA = Best Practice Alert; BMI = Body Mass index

© 2023 Simione M et al. *JAMA Network Open.*

eTable 2. Survey Results Evaluating Family's Experiences With the Connect for Health Program

	Denver Health	MGH	Prisma Health
	N=300	N=300	N=202
**Child, Parent and Household Characteristics**	n(%)	n(%)	n(%)
Race/Ethnicity			
Hispanic/Latino	258 (86.0)	233 (77.9)	66 (35.5)
White, Non-Hispanic	15 (5.0)	29 (9.7)	82 (44.1)
Black or African American, Non-Hispanic	19 (6.3)	22 (7.4)	34 (18.3)
American Indian/ Alaska Native, Native Hawaiian/ Pacific Islander, Asian	8 (2.7)	15 (5.0)	4 (2.1)
Education			
High school graduate or less	251 (83.9)	171 (57.6)	110 (61.5)
More than high school	48 (16.1)	126 (42.4)	69 (38.5)
Income			
Less than $20,000 yearly	88 (29.4)	90 (34.2)	47 (32.6)
$20,001 to $50,000 yearly	197 (65.9)	103 (39.2)	60 (41.7)
Greater than $50,000 yearly	14 (4.7)	70 (26.6)	37 (25.7)
**Impacts of the COVID-19 Pandemic**	n(%)	n(%)	n(%)
Compared to before the COVID-19 outbreak, how much is your child now doing the following:			
Eating			
Less	3 (1.0)	46 (15.7)	26 (13.1)
Same amount	91 (30.5)	161 (54.9)	109 (55.1)
More	204 (68.5)	86 (29.4)	63 (31.8)
Sleeping			
Less	36 (12.0)	41 (14.0)	19 (9.7)
Same amount	194 (64.9)	212 (72.6)	137 (69.9)
More	69 (23.1)	39 (13.4)	40 (20.4)
Physical activity			
Less	196 (65.6)	78 (26.7)	41 (20.7)
Same amount	100 (33.4)	107 (36.6)	86 (43.4)
More	3 (1.0)	107 (36.6)	71 (35.9)
Spending time outside			

© 2023 Simione M et al. *JAMA Network Open.*

Less	210 (70.7)	100 (34.2)	43 (21.7)
Same amount	85 (28.6)	82 (28.1)	72 (36.4)
More	2 (0.7)	110 (37.7)	83 (41.9)
Spending time watching TV, playing video/computer games, or using social media for educational purposes			
Less	3 (1.0)	72 (25.0)	18 (9.3)
Same amount	85 (29.3)	140 (48.6)	127 (65.5)
More	202 (69.7)	76 (26.4)	49 (25.3)
Spending time watching TV, playing video/computer games, or using social media for non-educational purposes			
Less	6 (2.1)	103 (35.5)	28 (14.3)
Same amount	96 (33.4)	115 (39.7)	128 (65.3)
More	185 (64.5)	72 (24.8)	40 (20.4)

Note. Dates of survey completion were as follows. Denver Health: 2/26/2021 – 9/2/2021; MGH: 10/14/2021 – 8/19/2022; Prisma Health: 7/16/2021 – 6/20/2022

© 2023 Simione M et al. *JAMA Network Open.*

eTable 3. Segmented Regression Model of Body Mass Index (BMI) *Z* Score Changes by BMI Percentiles in 5 Percentile Increments of Organizations Implementing Connect for Health

	Average BMI z-score change at 12-months	Average BMI z-score change at 24-months
**Initial BMI percentile category**	**Estimate (95% CI)**	**Estimate (95% CI)**
**Denver Health**		
**Children not eligible for the program (BMI between the 50-85th percentile) (N=1720)**		
50-54.9th percentile	**0.21 (0.17, 0.25), p<0.0001**	NA
55-59.9th percentile	**0.12 (0.09, 0.15), p<0.0001**	NA
60-64.9th percentile	**0.06 (0.02, 0.10), p=0.001**	NA
65-69.9th percentile	0.02 (-0.02, 0.06), p=0.35	NA
70-74.9th percentile	**-0.05 (-0.09, -0.01), p=0.03**	NA
75-79.9th percentile	**-0.15 (-0.20, -0.11), p<0.0001**	NA
80-84.9th percentile	**-0.25 (-0.29, -0.21), p<0.0001**	NA
**Children eligible for the program (BMI ≥ 85th) (N=1599)**		
85-89.9th percentile	**0.26 (0.22, 0.29), p<0.0001**	NA
90-94.9th percentile	**0.16 (0.13, 0.19), p<0.0001**	NA
95-98.9th percentile	**0.13 (0.11, 0.15), p<0.0001**	NA
99-100th percentile	**0.11 (0.06, 0.16), p<0.0001**	NA
**MGH**		
**Children not eligible for the program (BMI between the 50-85th percentile) (N=945)**		
50-54.9th percentile	**0.24 (0.16, 0.31), p<0.001**	**0.22 (0.12, 0.32), p<0.001**
55-59.9th percentile	**0.18 (0.08, 0.28), p=0.001**	**0.14 (0.05, 0.24), p=0.003**
60-64.9th percentile	0.07 (-0.01, 0.15), p=0.09	0.02 (-0.06, 0.11), p=0.63
65-69.9th percentile	**0.19 (0.11, 0.27), p<0.001**	**0.21 (0.13, 0.28), p<0.001**
70-74.9th percentile	**0.08 (0.01, 0.15), p=0.02**	0.04 (-0.03, 0.11), p=0.28
75-79.9th percentile	**0.17 (0.11, 0.24), p<0.001**	**0.12 (0.05, 0.19), p=0.001**
80-84.9th percentile	**0.07 (0.01, 0.13), p=0.03**	-0.004 (-0.07, 0.06), p=0.91
**Children eligible for the program (BMI ≥ 85th) (N=514)**		
85-89.9th percentile	**0.15 (0.01, 0.29), p=0.04**	**0.15 (0.01, 0.29), p=0.04**
90-94.9th percentile	**0.16 (0.10, 0.21), p<0.001**	**0.13 (0.06, 0.20), p<0.001**

© 2023 Simione M et al. *JAMA Network Open.*

95-98.9th percentile	**0.17 (0.14, 0.20), p<0.001**	**0.10 (0.06, 0.14), p<0.001**
99-100th percentile	**0.22 (0.15, 0.29), p<0.001**	0.07 (-0.02, 0.16), p=0.14
**Prisma Health**		
**Children not eligible for the program (BMI between the 50-95th percentile) (N=1604)**		
50-54.9th percentile	**0.14 (0.05, 0.23), p=0.002**	0.02 (-0.08, 0.12), p=0.74
55-59.9th percentile	**0.12 (0.03, 0.21), p=0.01**	0.07 (-0.03, 0.18), p=0.16
60-64.9th percentile	**0.13 (0.07, 0.20), p<0.001**	**0.10 (0.02, 0.17), p=0.01**
65-69.9th percentile	**0.13 (0.07, 0.18), p<0.001**	0.03 (-0.05, 0.12), p=0.39
70-74.9th percentile	**0.10 (0.05, 0.15), p=0.0003**	0.003 (-0.06, 0.07), p=0.92
75-79.9th percentile	0.05 (-0.005, 0.11), p=0.07	-0.05 (-0.12, 0.02), p=0.18
80-84.9th percentile	0.02 (-0.03, 0.06), p=0.51	-0.02 (-0.08, 0.04), p=0.51
85-89.9th percentile	-0.01 (-0.05, 0.03), p=0.59	**-0.10 (-0.15, -0.05), p=0.0001**
90-94.9th percentile	**-0.07 (-0.11, -0.03), p=0.001**	**-0.13 (-0.18, -0.09), p<0.001**
**Children eligible for the program (BMI ≥ 95th) (N=776)**		
95-98.9th percentile	**0.10 (0.08, 0.12), p<0.001**	**0.04 (0.01, 0.07), p=0.01**
99-100th percentile	0.05 (-0.01, 0.11), p=0.09	-0.04 (-0.11, 0.03), p=0.25

Note. BMI = Body mass index; CI = Confidence Interval. MGH and Prisma Health had extended implementation periods and Denver Health did not.

© 2023 Simione M et al. *JAMA Network Open.*

Supplemental Table 4. Segmented Regression Model of Body Mass Index (BMI) *Z* Score Changes By BMI Percentiles in 5 Percentile Increments of Geographically and Demographically Matched Control Organizations

	**Average BMI z-score change at 12-months**	**Average BMI z-score change at 24-months**
**Initial BMI percentile category**	**Estimate (95% CI)**	**Estimate (95% CI)**
**Control Organization for Denver Health**		
**Children with BMI between 50-85th percentile (N=339)**		
50-54.9th percentile	**0.33 (0.15, 0.51), p<0.001**	NA
55-59.9th percentile	**0.27 (0.16, 0.37), p<0.001**	NA
60-64.9th percentile	0.08 (-0.12, 0.28), p=0.42	NA
65-69.9th percentile	**0.23 (0.06, 0.39), p=0.010**	NA
70-74.9th percentile	**0.17 (0.03, 0.31), p=0.02**	NA
75-79.9th percentile	**0.21 (0.11, 0.31), p<0.001**	NA
80-84.9th percentile	0.07 (-0.04, 0.18), p=0.20	NA
**Children with BMI ≥ 85th percentile (N=344)**		
85-89.9th percentile	**0.23 (0.17, 0.29), p<0.001**	NA
90-94.9th percentile	**0.08 (0.02, 0.14), p<0.001**	NA
95-98.9th percentile	**0.10 (0.06, 0.15), p<0.001**	NA
99-100th percentile	**0.22 (0.07, 0.37), p=0.005**	NA
**Control Organization for MGH**		
**Children with BMI between 50-85th percentile (N=103)**		
50-54.9th percentile	-0.06 (-0.47, 0.35), p=0.73	0.002 (-0.52, 0.53), p=0.99
55-59.9th percentile	**0.27 (0.03, 0.50), p=0.03**	0.24 (-0.09, 0.57), p=0.14
60-64.9th percentile	0.21 (-0.04, 0.47), p=0.09	-0.03 (-0.39, 0.32), p=0.84
65-69.9th percentile	-0.01 (-0.19, 0.17), p=0.91	0.03 (-0.20, 0.26), p=0.78
70-74.9th percentile	0.03 (-0.21, 0.27), p=0.79	0.02 (-0.30, 0.34), p=0.91
75-79.9th percentile	**0.38 (0.13, 0.63), p=0.006**	**0.45 (0.19, 0.71), p=0.003**
80-84.9th percentile	0.11 (-0.12, 0.35), p=0.33	0.07 (-0.23, 0.37), p=0.62
**Children with BMI ≥ 85th percentile (N=148)**		
85-89.9th percentile	**0.24 (0.09, 0.39), p=0.003**	0.11 (-0.19, 0.41), p=0.45
90-94.9th percentile	**0.19 (0.03, 0.35), p=0.02**	0.08 (-0.09, 0.25), p=0.34

© 2023 Simione M et al. *JAMA Network Open.*

95-98.9th percentile	**0.10 (0.01, 0.19), p=0.03**	0.01 (-0.08, 0.10), p=0.80
99-100th percentile	0.08 (-0.07, 0.23), p=0.28	0.004 (-0.20, 0.21), p=0.97
**Control Organization for Prisma Health**		
**Children with BMI between 50-95th percentile (N=161)**		
50-54.9th percentile	-0.02 (-0.14, 0.11), p=0.80	-0.03 (-0.35, 0.29), p=0.86
55-59.9th percentile	0.02 (-0.45, 0.48), p=0.93	-0.51 (-1.37, 0.35), p=0.21
60-64.9th percentile	-0.10 (-0.38, 0.18), p=0.44	0.02 (-0.38, 0.41), p=0.92
65-69.9th percentile	0.13 (-0.21, 0.47), p=0.41	-0.08 (-0.38, 0.23), p=0.60
70-74.9th percentile	0.12 (-0.10, 0.34), p=0.26	-0.08 (-0.35, 0.18), p=0.53
75-79.9th percentile	0.25 (-0.06, 0.56), p=0.11	-0.17 (-0.49, 0.14), p=0.26
80-84.9th percentile	0.002 (-0.19, 0.20), p=0.98	0.09 (-0.15, 0.33), p=0.43
85-89.9th percentile	0.12 (-0.03, 0.27), p=0.12	0.06 (-0.18, 0.30), p=0.62
90-94.9th percentile	0.05 (-0.10, 0.20), p=0.51	-0.08 (-0.26, 0.09), p=0.34
**Children with BMI ≥ 95th percentile (N=66)**		
95-98.9th percentile	0.09 (-0.03, 0.20), p=0.13	0.06 (-0.09, 0.20), p=0.44
99-100th percentile	0.09 (-0.04, 0.22), p=0.16	0.09 (-0.07, 0.24), p=0.28

Note. BMI = Body mass index; CI = Confidence Interval. MGH and Prisma Health had extended implementation periods and therefore we examined BMI change at 24 months for organizations serving as controls.

© 2023 Simione M et al. *JAMA Network Open.*
