# Supplementary material for: Evaluating the Implementation of the Connect for Health Pediatric Weight Management Program
Source: JAMA Netw Open. 2024 Jan 25;7(1):e2352648. doi: 10.1001/jamanetworkopen.2023.52648 (PMC10811559; doi:10.1001/jamanetworkopen.2023.52648)
Supplement: Supplement 2. — Data Sharing Statement [file jamanetwopen-e2352648-s002.pdf]

Data Sharing Statement

Simione. Evaluating the Implementation of the Connect for Health Pediatric Weight Management Program. *JAMA Netw Open*. Published January 25, 2024. doi:10.1001/jamanetworkopen.2023.52648

Data
Data available: No

Additional Information
Explanation for why data not available: Data will be made available upon reasonable request
